# The expanded Laboratory of Collaborative and Field Research

**DOI:** 10.1098/rstb.2008.4010

**Published:** 2008-11-27

**Authors:** Judith Farquhar

**Affiliations:** Department of Anthropology, University of Chicago1126 East 59th Street, Chicago, IL 60637, USA

Along with David Asher and Richard Benfante, who also attended the End of Kuru conference, I spoke there as a member of the wandering and far-flung community of Carleton Gajdusek's Bethesda Laboratory at the National Institutes of Health (NIH). I worked there, originally talking my way into a clerical job, despite having no particular qualifications, as a ‘social science analyst’ from 1967 to the mid-1970s; I tend to think of my job description throughout, however, as ‘handmaiden’. For years I worked on whatever came to hand, or whatever was thrust into my hands by Carleton. After I left the laboratory to go to graduate school in anthropology, I edited with Carleton the volume called *Kuru: Correspondence and Field Notes from the Collection of D. Carleton Gajdusek*. Since then I have been working on health and medicine in China as a social anthropologist, teaching and doing research in Chicago, North Carolina and Beijing.

Like many nostalgic historians and travellers who can claim many homes, like Carleton, even, I have a very partial, very personally motivated version of that place and time. Still, because Carleton, kuru, its scientists and the laboratory had such an important role in my own formation as a person, it interests me to ask: ‘What was kuru from the point of view of the laboratory? What kind of a place was the laboratory?’ My answers to these questions have become broader and vaguer over the years but my interest in understanding this past is no less intense than it ever was.

## 1. The laboratory

We used to answer the phone, ‘Dr Gajdusek's Office’, though the formal name was the Laboratory of Collaborative and Field Research. This moniker, which both over- and understated the mission of the research unit, signalled both the ambitions Carleton had for his own laboratory and the character it increasingly took on in the course of the 1960s, 1970s and 1980s. This laboratory was both a small set of physical sites, including (at times) two suites of rooms at NIH, a building at Fort Detrick and various animal facilities, and a node in a network of research activities. It would not be amiss to see the network itself, vast as it was, as both ‘Dr Gajdusek's Office’ and the ‘Laboratory of Collaborative and Field Research’. The global coordination of kuru research, at least in the early years, could not be confined to the laboratory benches, file cabinets, photo archives and specimen collections of just a few sites in the USA. It could not even be limited to the community of scientists whose relations with each other—ranging from romances to resentments (and sometimes including both)—were so dependent on the work of the laboratory. I seem to recall periods of time in which Carleton had to fiercely defend to the NIH administration his conception of the work of our unit as collaborative and field based. The good scientific reasons why it was imperative to fund international travel for scientists (and, in the case of the Alpha Helix expedition, even handmaidens), as well as to support and transport, sometimes halfway around the world, field collections ranging from blood specimens to cinema films, had to be spelled out again and again.

As long as this vision of the scientific project succeeded, it had interesting results for those of us who mostly stayed in Bethesda. The Laboratory of Collaborative and Field Research accumulated an ever-thicker global network, instantiated in Carleton's ‘Family and Friends’ list that for a time I helped to compulsively maintain. There were visiting investigators who, once added to the Bethesda community, became permanent members of the global family and friends community. There were frequent marathon mailings of reprints when all staff, no matter how lofty, gathered to stuff envelopes with recent publications for worldwide dissemination. There were dinners at Carleton's house in which teenage cooks rustled up huge meals for unpredictable numbers of globetrotting scientists, usually invited on the spur of the moment. There were the precious Revco freezers in which a phenomenal variety of specimens waited to be useful, the freezers themselves demanding meticulous record keeping from us. There were the cinema and still photographic records from Papua New Guinea (PNG), Micronesia and other parts of the Pacific, all requiring assembly, copying, documenting and special storage. And there were Carleton's journals, growing all the time with descriptions of other fields, laboratories, scientists, museums, villages, mountain trails and palaces; bursting with analyses of everything from research reports to the author's own motives; speculations and hypotheses; facts historical, natural historical, metaphysical and geographical; gossip and scandal; and embarrassing psychoanalysis of all of us.

## 2. Kuru itself

The ambitions of the Bethesda laboratory to collect and to archive, far beyond anyone's capacity to process the collections into useful knowledge, were far vaster than the crowded spaces in which experiments, observations, writing and publishing got done. The outsized world that flowed through these few rooms is a testament to a certain refusal to reduce the import of kuru and its kindred diseases. Though this network was a laboratory of sorts, the variables of the phenomena being studied were not under control. Eastern Highlanders who made kuru research possible in PNG were important presences in the laboratory via writing and film; scientists and data from other laboratories that undertook key collaborative experiments were frequent visitors; kuru existed and travelled in field specimens of frozen tissue, in tissue culture, and in animals that travelled through Bethesda, Frederick, Patuxent and Gulf South; anthropologists gathered demographic, cultural and practical information about what we called ‘the kuru region’ from the multidimensional archive in the laboratories; and reports, analyses and speculations about the biological significance of kuru got put together, torn apart, put together again, and finally published to eventually form their own diaspora from Bethesda. Despite its rigorously local character as a human epidemic, kuru after 1957 quickly became a global phenomenon, and the Bethesda laboratory, handmaidens and all, was one of its key nodes. So many people who were not able to gather in London in 2007 were part of that project and made it work—they too are part of the far-flung kuru laboratory. Given the global reach of kuru, and the long memories and archived lives so many of us have, perhaps we cannot really declare an end to kuru at all.

